# The Top 100 Most Cited Journal Articles on Hydrocephalus

**DOI:** 10.7759/cureus.54481

**Published:** 2024-02-19

**Authors:** Laurel A Seltzer, Mitchell W Couldwell, R. Shane Tubbs, CJ Bui, Aaron S Dumont

**Affiliations:** 1 Department of Neurosurgery, Tulane University School of Medicine, New Orleans, USA; 2 Department of Anatomical Sciences, St. George's University, St. George's, GRD; 3 Department of Neurosurgery and Ochsner Neuroscience Institute, Ochsner Health System, New Orleans, USA; 4 Department of Structural Biology, Tulane University School of Medicine, New Orleans, USA

**Keywords:** neurosurgery, pediatrics neurosurgery, journal citation report, citation analysis, pediatric hydrocephalus, bibliometric analysis, shunt, hydrocephalus

## Abstract

Hydrocephalus represents a significant burden of disease, with more than 383,000 new cases annually worldwide. When the magnitude of this condition is considered, a centralized archive of pertinent literature is of great clinical value. From a neurosurgical standpoint, hydrocephalus is one of the most frequently treated conditions in the field. The focus of this study was to identify the top 100 journal articles specific to hydrocephalus using bibliometric analysis. Using the Journal of Citation Report database, 10 journals were identified. The Web of Science Core Collection was then searched using each journal name and the search term “hydrocephalus.” The results were ordered by “Times Cited” and searched by the number of citations. The database contained journal articles from 1976 to 2021, and the following variables were collected for analysis: journal, article type, year of publication, and the number of citations. Journal articles were excluded if they had no relation to hydrocephalus, mostly involved basic science research, or included animal studies. Ten journals were identified using the above criteria, and a catalog of the 100 most cited publications in the hydrocephalus literature was created. Articles were arranged from highest to lowest citation number, with further classification by journal, article type, and publication year. Of the 100 articles referenced, 38 were review articles, 24 were original articles, 15 were comparative studies, 11 were clinical trials, six were multi-center studies, three were cross-sectional, and three were case reports with reviews. Articles were also sorted by study type and further stratified by etiology. If the etiology was not specified, studies were instead subcategorized by treatment type. Etiologies such as aqueductal stenosis, tumors, and other obstructive causes of hydrocephalus were classified as obstructive (n=6). Communicating (n=15) included idiopathic, normal pressure hydrocephalus, and other non-obstructive etiologies. The category “other” (n=3) was assigned to studies that included etiologies, populations, and/or treatments that did not fit into the classifications previously outlined. Through our analysis of highly cited journal articles focusing on different etiologies and the surgical or medical management of hydrocephalus, we hope to elucidate important trends. By establishing the 100 most cited hydrocephalus articles, we contribute one source, stratified for efficient referencing, to facilitate clinical care and future research on hydrocephalus.

## Introduction and background

Hydrocephalus is the pathologic accumulation of excess cerebrospinal fluid (CSF) within the calvaria, brain parenchyma, or intraventricularly [1]. The divergence in anatomical designation among existing literature can be further elucidated with the following definition: "Hydrocephalus is an active distension of the ventricular system of the brain resulting from inadequate passage of CSF from its point of production within the cerebral ventricles to its point of absorption into the systemic circulation" [2]. This broader classification mirrors the variation in the clinical picture of hydrocephalus. On presentation, patients can assume an array of symptoms, with underlying etiologies including increased CSF production, decreased CSF absorption, or obstructed flow from the ventricles to the subarachnoid space [1-3]. This great variability can elicit further complexity for clinicians and researchers managing and studying this condition.

Many journal articles have been published about hydrocephalus. One way to analyze the impact of these publications is to use citation analysis. Bibliometric analyses using online databases such as the Web of Science Core Collection have systematized the influx of medical and biological publications during this information era [4,5]. The earliest database to track citations was developed in 1962 by the Institute for Science Information, which was later combined with the Social Sciences Index in 1973 and the Arts and Humanities Citation Index in 1978 to create a comprehensive database. This merged database was presented online in 1997 under the name Web of Science, which would later become the Web of Science Core Collection [6]. Recent publications have evaluated relevant work in different fields through citation analysis. For example, articles summarizing the most cited research in pediatric neurosurgery, orthopedic surgery, urethral reconstruction, epilepsy, and status epilepticus have contributed to the literature [6-10]. In this review, we present the 100 most highly cited journal articles about hydrocephalus published in ten journals, the citations being gathered from the Web of Science Core Collection.

## Review

Methods

**Figure 1:**
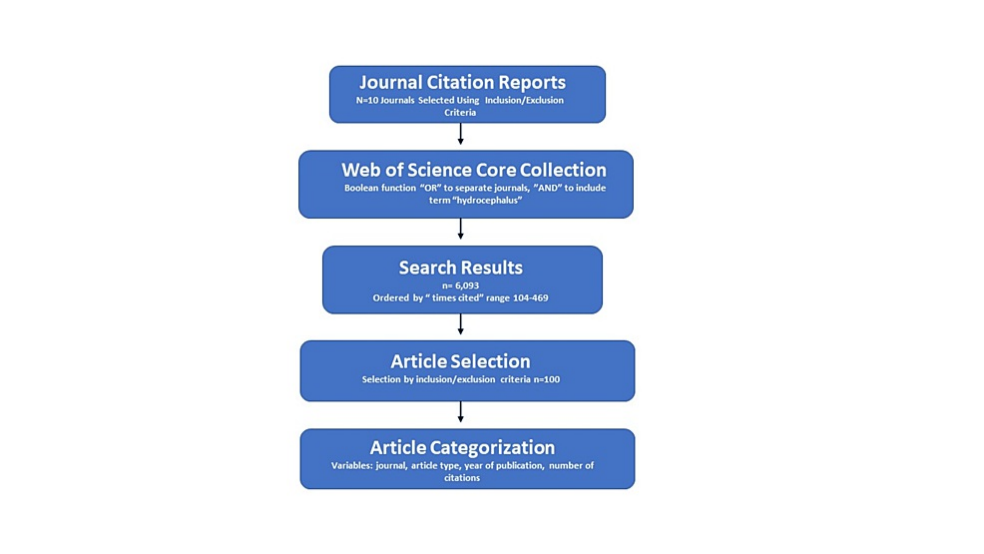
Chart depicting journal selection and search criteria

The focus of this study was to identify journal articles specifically dedicated to hydrocephalus. Using the same methods as Ponce and Lozano and later Grayson et al., 10 journals were identified using the Journal of Citation Report [6,7]. The database was searched using the terms “neurosurgery,” “neurological surgery,” “pediatric neurosurgery,” and “pediatric neurological surgery.” These journals were: Child’s Brain, Child’s Nervous System, Journal of Neurosurgery, Journal of Neurosurgery Pediatrics, Journal of Pediatrics, Neuropediatrics, Neurosurgery, Pediatric Neurosurgery, Pediatrics, and World Neurosurgery. Once they were identified, the Web of Science Core Collection was searched using each journal name and the Boolean function “OR” to separate the journals, and the Boolean function “AND” to include the search term “hydrocephalus.” The results were then ordered by “Times Cited,” which included all the results from the 10 journals searched by the number of citations per journal article. The database contained journal articles from 1976 to 2021, and the following variables were collected for analysis: journal, article type, year of publication, and the number of citations. Journal articles were excluded if they had no relation to hydrocephalus, were based on basic science research, or included animal studies (Figure 1).

Results

Sources and Citations

Ten journals were identified (Table 1) using the selection criteria outlined above, and articles were sorted from highest to lowest in citation number, with the search returning 1,554 results. The impact factor of the journals identified in the Journal of Citation Reports ranged from 0.985 to 7.124. Thirty-six percent of articles were published in journals with an impact factor greater than 5, while 52% were found in journals with an impact factor of 1-5, and 12% were found in journals with an impact factor under 1. Among the 100 journal articles selected, 33 were published in the Journal of Neurosurgery, 31 in Neurosurgery, 12 in Pediatric Neurosurgery, nine each in the Journal of Neurosurgery Pediatrics and Child’s Nervous System, three in Pediatrics, two in the Journal of Pediatrics, one in World Neurosurgery, and none in either Neuropediatrics or Child’s Brain. The 100 most cited papers on hydrocephalus were then listed (Table 2), ranked from highest to lowest number of citations. The article name, associated journal, and year of publication were listed as well.

**Table 1:** Impact factors of selected journals

Journal	Impact Factor	Number of Journal Articles
Child's Brain	0.985	0
Child's Nervous System	1.475	9
Journal of Neurosurgery	5.115	33
Journal of Neurosurgery Pediatrics	2.375	9
Journal of Pediatrics	4.113	2
Neuropediatrics	1.947	0
Neurosurgery	3.968	31
Pediatric Neurosurgery	0.985	12
Pediatrics	7.124	3
World Neurosurgery	1.829	1

**Table 2:** The 100 most cited papers on hydrocephalus

Rank	Citations	Article	Journal	Year of Publication
1	469	Drake JM, Kestle JRW, Milner R, et al. Randomized trial of cerebrospinal fluid shunt valve design in pediatric hydrocephalus. Neurosurgery. 1998;43(2):294-303. doi:10.1097/00006123-199808000-00068 [11]	Neurosurgery	1998
2	375	Hopf NJ, Grunert P, Fries G, Resch KDM, Perneczky A. Endoscopic third ventriculostomy: outcome analysis of 100 consecutive procedures. Neurosurgery. Apr 1999;44(4):795-804. doi:10.1097/00006123-199904000-00062 [12]	Neurosurgery	1999
3	317	Vangijn J, Hijdra A, Wijdicks EFM, Vermeulen M, Vancrevel H. Acute hydrocephalus after aneurysmal subarachnoid hemorrhage. Journal of Neurosurgery. 1985;63(3):355-362. doi:10.3171/jns.1985.63.3.0355 [13]	Journal of Neurosurgery	1985
4	303	Sainterose C, Piatt JH, Renier D, et al. Mechanical complications in shunts. Pediatric Neurosurgery. 1992;17(1):2-9. doi:10.1159/000120557 [14]	Pediatric Neurosurgery	1992
5	292	Hebb AO, Cusimano MD. Idiopathic normal, pressure hydrocephalus: a systematic review of diagnosis and outcome. Neurosurgery. Nov 2001;49(5):1166-1184. doi:10.1097/00006123-200111000-00028 [15]	Neurosurgery	2001
6	271	Kestle J, Drake J, Milner R, et al. Long-term follow-up data from the shunt design trial. Article. Pediatric Neurosurgery. Nov 2000;33(5):230-236. doi:10.1159/000055960 [16]	Pediatric Neurosurgery	2000
7	266	Choux M, Genitori L, Lang D, Lena G. Shunt implantation – reducing the incidence of shunt infection. Journal of Neurosurgery. Dec 1992;77(6):875-880. doi:10.3171/jns.1992.77.6.0875 [17]	Journal of Neurosurgery	1992
8	263	Relkin N, Marmarou A, Klinge P, Bergsneider M, Black PM. Diagnosing idiopathic normal-pressure hydrocephalus. Neurosurgery. Sep 2005;57(3):4-16. doi:10.1227/01.Neu.0000168185.29659.C5 [18]	Neurosurgery	2005
9	243	Marmarou A, Bergsneider M, Klinge P, Relkin N, Black PM. The value of supplemental prognostic tests for the preoperative assessment of idiopathic normal-pressure hydrocephalus. Neurosurgery. Sep 2005;57(3):17-28. doi:10.1227/01.Neu.0000168184.01002.60 [19]	Neurosurgery	2005
10	240	Kulkarni AV, Drake JM, Lamberti-Pasculli M. Cerebrospinal fluid shunt infection: a prospective study of risk factors. Journal of Neurosurgery. Feb 2001;94(2):195-201. doi:10.3171/jns.2001.94.2.0195 [20]	Journal of Neurosurgery	2001
11	236	Cinalli G, Sainte-Rose C, Chumas P, et al. Failure of third ventriculostomy in the treatment of aqueductal stenosis in children. Journal of Neurosurgery. Mar 1999;90(3):448-454. doi:10.3171/jns.1999.90.3.0448 [21]	Journal of Neurosurgery	1999
12	230	Tuli S, Drake J, Lawless J, Wigg M, Lamberti-Pasculli M. Risk factors for repeated cerebrospinal shunt failures in pediatric patients with hydrocephalus. Journal of Neurosurgery. Jan 2000;92(1):31-38. doi:10.3171/jns.2000.92.1.0031 [22]	Journal of Neurosurgery	2000
13	225	Schroeder HWS, Niendorf WR, Gaab MR. Complications of endoscopic third ventriculostomy. Journal of Neurosurgery. Jun 2002;96(6):1032-1040. doi:10.3171/jns.2002.96.6.1032 [23]	Journal of Neurosurgery	2002
14	221	Boon AJW, Tans JTJ, Delwel EJ, et al. Dutch Normal-Pressure Hydrocephalus Study. Prediction of outcome after shunting by resistance to outflow of cerebrospinal fluid. Journal of Neurosurgery. Nov 1997;87(5):687-693. doi:10.3171/jns.1997.87.5.0687 [24]	Journal of Neurosurgery	1997
15	217	Patwardhan RV, Nanda A. Implanted ventricular shunts in the United States: The billion-dollar-a-year cost of hydrocephalus treatment. Neurosurgery. Jan 2005;56(1):139-144. doi:10.1227/01.Neu.0000146206.40375.41 [25]	Neurosurgery	2005
16	214	O'Hayon BB, Drake JM, Ossip MG, Tuli S, Clarke M. Frontal and occipital horn ratio: A linear estimate of ventricular size for multiple imaging modalities in pediatric hydrocephalus. Pediatric Neurosurgery. Nov 1998;29(5):245-249. doi:10.1159/000028730 [26]	Pediatric Neurosurgery	1998
17	211	Black PM. Idiopathic normal-pressure hydrocephalus – results of shunting in 62 patients. Journal of Neurosurgery. 1980;52(3):371-377. doi:10.3171/jns.1980.52.3.0371[27]	Journal of Neurosurgery	1980
18	205	Marmarou A, Young HF, Aygok GA, et al. Diagnosis and management of idiopathic normal-pressure hydrocephalus: a prospective study in 151 patients. Journal of Neurosurgery. Jun 2005;102(6):987-997. doi:10.3171/jns.2005.102.6.0987 [28]	Journal of Neurosurgery	2005
19	200	Wu Y, Green NL, Wrensch MR, Zhao SJ, Gupta N. Ventriculoperitoneal shunt complications in California: 1990 to 2000. Neurosurgery. Sep 2007;61(3):557-562. doi:10.1227/01.Neu.0000290903.07943.Af [29]	Neurosurgery	2007
20	195	Teo C, Jones R. Management of hydrocephalus by endoscopic third ventriculostomy in patients with myelomeningocele. Pediatric Neurosurgery. Aug 1996;25(2):57-63. doi:10.1159/000121098 [30]	Pediatric Neurosurgery	1996
21	193	Dorai Z, Hynan LS, Kopitnik TA, Samson D. Factors related to hydrocephalus after aneurysmal subarachnoid hemorrhage. Neurosurgery. Apr 2003;52(4):763-769. doi:10.1227/01.Neu.0000053222.74852.2d [31]	Neurosurgery	2003
22	193	Fukuhara T, Vorster SJ, Luciano MG. Risk factors for failure of endoscopic third ventriculostomy for obstructive hydrocephalus. Neurosurgery. May 2000;46(5):1100-1109. doi:10.1097/00006123-200005000-00015 [32]	Neurosurgery	2000
23	193	Keucher TR, Mealey J. Long-term results after ventriculoatrial and ventriculoperitoneal shunting for infantile hydrocephalus. Journal of Neurosurgery. 1979;50(2):179-186. doi:10.3171/jns.1979.50.2.0179 [33]	Journal of Neurosurgery	1979
24	192	Warf BC. Comparison of endoscopic third ventriculostomy alone and combined with choroid plexus cauterization in infants younger than 1 year of age: a prospective study in 550 African children. Journal of Neurosurgery. Dec 2005;103(6):475-481. doi:10.3171/ped.2005.103.6.0475 [34]	Journal of Neurosurgery	2005
25	189	Brockmeyer D, Abtin K, Carey L, Walker ML. Endoscopic third ventriculostomy: An outcome analysis. Pediatric Neurosurgery. May 1998;28(5):236-240. doi:10.1159/000028657 [35]	Pediatric Neurosurgery	1998
26	185	Piatt JH, Carlson CV. A search for determinants of cerebrospinal-fluid shunt survival – retrospective analysis of a 14-year institutional experience. Pediatric Neurosurgery. Sep-Oct 1993;19(5):233-242. doi:10.1159/000120738 [36]	Pediatric Neurosurgery	1993
27	183	Simon TD, Riva-Cambrin J, Srivastava R, et al. Hospital care for children with hydrocephalus in the United States: utilization, charges, comorbidities, and deaths. Journal of Neurosurgery-Pediatrics. Feb 2008;1(2):131-137. doi:10.3171/ped/2008/1/2/131 [37]	Journal of Neurosurgery-Pediatrics	2008
28	182	Kulkarni AV, Drake JM, Mallucci CL, et al. Endoscopic third ventriculostomy in the treatment of childhood hydrocephalus. Journal of Pediatrics. Aug 2009;155(2):254-259. doi:10.1016/j.jpeds.2009.02.048 [38]	Journal of Pediatrics	2009
29	181	Dirocco C, Marchese E, Velardi F. A survey of the 1^st^ complication of newly implanted CSF shunt devices for the treatment of nontumoral hydrocephalus – cooperative survey of the 1991-1992 education committee of the ISPN. Childs Nervous System. Jul 1994;10(5):321-327. doi:10.1007/bf00335171 [39]	Childs Nervous System	1994
30	180	Pollack IF, Albright AL, Adelson PD, Hakim-Medos Investigator G. A randomized, controlled study of a programmable shunt valve versus a conventional valve for patients with hydrocephalus. Neurosurgery. Dec 1999;45(6):1399-1408. doi:10.1097/00006123-199912000-00026 [40]	Neurosurgery	1999
31	179	Warf BC. Hydrocephalus in Uganda: the predominance of infectious origin and primary management with endoscopic third ventriculostomy. Journal of Neurosurgery. Jan 2005;102(1 Suppl):1-15. Doi:10.3171/peds.2005.102.1.0001 [41]	Journal of Neurosurgery	2005
32	175	Bondurant CP, Jimenez DF. Epidemiology of cerebrospinal fluid shunting. Article. Pediatric Neurosurgery. Nov 1995;23(5):254-258. doi:10.1159/000120968 [42]	Journal of Neurosurgery	1995
33	172	Pople IK, Bayston R, Hayward RD. Infection of cerebrospinal-fluid shunts in infants – a study of etiologic factors. Article. Journal of Neurosurgery. Jul 1992;77(1):29-36. doi:10.3171/jns.1992.77.1.0029 [43]	Journal of Neurosurgery	1992
34	170	McLaughlin MR, Wahlig JB, Kaufmann AM, Albright AL. Traumatic basilar aneurysm after endoscopic third ventriculostomy: case report. Article. Neurosurgery. Dec 1997;41(6):1400-1403. doi:10.1097/00006123-199712000-00034 [44]	Neurosurgery	1997
35	167	Buxton N, Macarthur D, Malucci C, Punt J, Vloeberghs M. Neuroendoscopic third ventriculostomy in patients less than 1 year old. Article. Pediatric Neurosurgery. Aug 1998;29(2):73-76. doi:10.1159/000028693 [45]	Pediatric Neurosurgery	1998
36	167	Boon AJW, Tans JTJ, Delwel EJ, et al. Dutch normal-pressure hydrocephalus study: randomized comparison of low- and medium-pressure shunts. Article. Journal of Neurosurgery. Mar 1998;88(3):490-495. doi:10.3171/jns.1998.88.3.0490 [46]	Journal of Neurosurgery	1998
37	165	Adams-Chapman I, Hansen NI, Stoll BJ, Higgins R, Network NR. Neurodevelopmental outcome of extremely low birth weight infants with posthemorrhagic hydrocephalus requiring shunt insertion. Article. Pediatrics. May 2008;121(5):E1167-E1177. doi:10.1542/peds.2007-0423 [47]	Pediatrics	2008
38	162	Siomin V, Cinalli G, Grotenhuis A, et al. Endoscopic third ventriculostomy in patients with cerebrospinal fluid infection and/or hemorrhage. Article. Journal of Neurosurgery. Sep 2002;97(3):519-524. doi:10.3171/jns.2002.97.3.0519 [48]	Journal of Neurosurgery	2002
39	162	Cinalli G, Salazar C, Mallucci C, Yada JZ, Zerah M, Sainte-Rose C. The role of endoscopic third ventriculostomy in the management of shunt malfunction. Article. Neurosurgery. Dec 1998;43(6):1323-1327. doi:10.1097/00006123-199812000-00030ment of shunt malfunction [49]	Neurosurgery	1998
40	161	McGirt MJ, Woodworth G, Coon AL, Thomas G, Williams MA, Rigamonti D. Diagnosis, treatment, and analysis of long-term outcomes in idiopathic normal-pressure hydrocephalus. Article. Neurosurgery. Oct 2005;57(4):699-705. doi:10.1227/01.Neu.0000175724.00147.10 [50]	Neurosurgery	2005
41	161	McGirt MJ, Leveque JC, Wellons JC, et al. Cerebrospinal fluid shunt survival and etiology of failures: a seven-year institutional experience. Article. Pediatric Neurosurgery. May 2002;36(5):248-255. doi:10.1159/000058428 [51]	Pediatric Neurosurgery	2002
42	161	Oka K, Yamamoto M, Hoffman HJ, Ikeda K, Tomonaga M, Kelly PJ. Flexible endoneurosurgical therapy for aqueductal stenosis. Article. Neurosurgery. Aug 1993;33(2):236-243. doi:10.1227/00006123-199308000-00009 [52]	Neurosurgery	1993
43	159	Eide PK, Sorteberg W. Diagnostic intracranial pressure monitoring and surgical management in idiopathic normal pressure hydrocephalus: A 6-Year Review of 214 Patients. Article. Neurosurgery. Jan 2010;66(1):80-90. doi:10.1227/01.Neu.0000363408.69856.B8 [53]	Neurosurgery	2010
44	157	Vale FL, Bradley EL, Fisher WS. The relationship of subarachnoid hemorrhage and the need for postoperative shunting. Article. Journal of Neurosurgery. Mar 1997;86(3):462-466. doi:10.3171/jns.1997.86.3.0462 [54]	Journal of Neurosurgery	1997
45	153	Kelly PJ. Stereotaxic 3^rd^ ventriculostomy in patients with nontumoral adolescent adult onset aqueductal stenosis and symptomatic hydrocephalus. Article. Journal of Neurosurgery. Dec 1991;75(6):865-873. doi:10.3171/jns.1991.75.6.0865 [55]	Journal of Neurosurgery	1991
46	151	Kestle JRW, Drake JM, Cochrane D, et al. Lack of benefit of endoscopic ventriculoperitoneal shunt insertion: a multicenter randomized trial. Article. Journal of Neurosurgery. Feb 2003;98(2):284-290. doi:10.3171/jns.2003.98.2.0284 [56]	Journal of Neurosurgery	2003
47	149	Reddy GK, Bollam P, Caldito G. Long-term outcomes of ventriculoperitoneal shunt surgery in patients with hydrocephalus. Article. World Neurosurgery. Feb 2014;81(2):404-410. doi:10.1016/j.wneu.2013.01.096 [57]	Journal of Neurosurgery	2014
48	149	Sainte-Rose C, Lacombe J, Pierrekahn A, Renier D, Hirsch JF. Intracranial venous sinus hypertension – cause or consequence of hydrocephalus in infants. Article. Journal of Neurosurgery. 1984;60(4):727-736. doi:10.3171/jns.1984.60.4.0727 [58]	Journal of Neurosurgery	1984
49	148	Sainte-Rose C, Cinalli G, Roux FE, et al. Management of hydrocephalus in pediatric patients with posterior fossa tumors: the role of endoscopic third ventriculostomy. Article. Journal of Neurosurgery. Nov 2001;95(5):791-797. doi:10.3171/jns.2001.95.5.0791 [59]	Journal of Neurosurgery	2001
50	143	Zemack G, Romner B. Seven years of clinical experience with the programmable Codman Hakim valve: a retrospective study of 583 patients. Article. Journal of Neurosurgery. Jun 2000;92(6):941-948. doi:10.3171/jns.2000.92.6.0941 [60]	Journal of Neurosurgery	2000
51	141	Simon TD, Hall M, Riva-Cambrin J, et al. Infection rates following initial cerebrospinal fluid shunt placement across pediatric hospitals in the United States. Article. Journal of Neurosurgery-Pediatrics. Aug 2009;4(2):156-165. doi:10.3171/2009.3.Peds08215 [61]	Journal of Neurosurgery-Pediatrics	2009
52	141	Feng HL, Huang GF, Liao XL, et al. Endoscopic third ventriculostomy in the management of obstructive hydrocephalus: an outcome analysis. Article. Journal of Neurosurgery. Apr 2004;100(4):626-633. doi:10.3171/jns.2004.100.4.0626 [62]	Journal of Neurosurgery	2004
53	140	Krauss JK, Droste DW, Vach W, et al. Cerebrospinal fluid shunting in idiopathic normal-pressure hydrocephalus of the elderly: effect of periventricular and deep white matter lesions. Article. Neurosurgery. Aug 1996;39(2):292-299. doi:10.1097/00006123-199608000-00011 [63]	Neurosurgery	1996
54	140	Vassilouthis J, Richardson AE. Ventricular dilatation and communicating hydrocephalus following spontaneous subarachnoid hemorrhage. Article. Journal of Neurosurgery. 1979;51(3):341-351. doi:10.3171/jns.1979.51.3.0341 [64]	Journal of Neurosurgery	1979
55	139	Waziri A, Fusco D, Mayer SA, McKhann GM, Connolly ES. Postoperative hydrocephalus in patients undergoing decompressive hemicraniectomy for ischemic or hemorrhagic stroke. Article. Neurosurgery. Sep 2007;61(3):489-493. doi:10.1227/01.Neu.0000290894.85072.37 [65]	Neurosurgery	2007
56	137	Stone JJ, Walker CT, Jacobson M, Phillips V, Silberstein HJ. Revision rate of pediatric ventriculoperitoneal shunts after 15 years. Clinical article. Article. Journal of Neurosurgery-Pediatrics. Jan 2013;11(1):15-19. doi:10.3171/2012.9.Peds1298 [66]	Journal of Neurosurgery-Pediatrics	2013
57	137	Handler MH, Abbott R, Lee M. A near-fatal complication of endoscopic 3^rd^ ventriculostomy – case report. Note. Neurosurgery. Sep 1994;35(3):525-527. doi:10.1227/00006123-199409000-00025 [67]	Neurosurgery	1994
58	134	Kestle JRW, Riva-Cambrin J, Wellons JC, et al. A standardized protocol to reduce cerebrospinal fluid shunt infection: the Hydrocephalus Clinical Research Network Quality Improvement Initiative. Clinical article. Article. Journal of Neurosurgery-Pediatrics. Jul 2011;8(1):22-29. doi:10.3171/2011.4.Peds10551 [68]	Journal of Neurosurgery-Pediatrics	2011
59	132	Luetmer PH, Huston J, Friedman JA, et al. Measurement of cerebrospinal fluid flow at the cerebral aqueduct by use of phase-contrast magnetic resonance imaging: technique validation and utility in diagnosing idiopathic normal pressure hydrocephalus. Article. Neurosurgery. Mar 2002;50(3):534-542. doi:10.1097/00006123-200203000-00020 [69]	Neurosurgery	2002
60	131	Aschoff A, Kremer P, Benesch C, Fruh K, Klank A, Kunze S. Overdrainage and shunt technology – a critical comparison of programmable, hydrostatic and variable resistance valves and flow-reducing devices. Article. Childs Nervous System. Apr 1995;11(4):193-202. doi:10.1007/bf00277653 [70]	Childs Nervous System	1995
61	127	Surgical Bergsneider M, Black PM, Klinge P, Marmarou A, Relkin N. Surgical management of idiopathic normal-pressure hydrocephalus. Article. Neurosurgery. Sep 2005;57(3):29-39. doi:10.1227/01.Neu.0000168186.45363.4dmanagement of idiopathic normal-pressure hydrocephalus [71]	Neurosurgery	2005
62	126	Govender ST, Nathoo N, van Dellen JR. Evaluation of an antibiotic-impregnated shunt system for the treatment of hydrocephalus. Article. Journal of Neurosurgery. Nov 2003;99(5):831-839. doi:10.3171/jns.2003.99.5.0831 [72]	Journal of Neurosurgery	2003
63	126	Sotelo J, Marin C. Hydrocephalus secondary to cysticercotic arachnoiditis – a long-term follow-up review of 92 cases. Article. Journal of Neurosurgery. May 1987;66(5):686-689. doi:10.3171/jns.1987.66.5.0686 [73]	Journal of Neurosurgery	1987
64	125	Hoffman HJ, Harwoodnash D, Gilday DL. Percutaneous 3^rd^ ventriculostomy in the management of noncommunicating hydrocephalus. Article. Neurosurgery. 1980;7(4):313-321. doi:10.1227/00006123-198010000-00002 [74]	Neurosurgery	1980
65	123	Gruber A, Reinprecht A, Bavinzski G, Czech T, Richling B. Chronic shunt-dependent hydrocephalus after early surgical and early endovascular treatment of ruptured intracranial aneurysms. Article. Neurosurgery. Mar 1999;44(3):503-509. doi:10.1097/00006123-199903000-00039 [75]	Neurosurgery	1999
66	123	Caldarelli M, DiRocco C, LaMarca F. Shunt complications in the first postoperative year in children with meningomyelocele. Article. Childs Nervous System. Dec 1996;12(12):748-754. doi:10.1007/bf00261592 [76]	Childs Nervous System	1996
67	123	Hill A, Volpe JJ. Decrease in pulsatile flow in the anterior cerebral-arteries in infantile hydrocephalus. Article. Pediatrics. 1982;69(1):4-7 [77]	Pediatrics	1982
68	122	Kulkarni AV, Drake JM, Kestle JRW, et al. Predicting who will benefit from endoscopic third ventriculostomy compared with shunt insertion in childhood hydrocephalus using the ETV success score clinical article. Article. Journal of Neurosurgery-Pediatrics. Oct 2010;6(4):310-315. doi:10.3171/2010.8.Peds103 [78]	Journal of Neurosurgery-Pediatrics	2010
69	122	Drake JM, Kestle JRW, Tuli S. CSF shunts 50 years on - past, present and future. Article; Proceedings Paper. Childs Nervous System. Nov 2000;16(10-11):800-804. doi:10.1007/s003810000351 [79]	Childs Nervous System	2000
70	121	Oi S, Di Rocco C. Proposal of "evolution theory in cerebrospinal fluid dynamics" and minor pathway hydrocephalus in developing immature brain. Article. Childs Nervous System. Jul 2006;22(7):662-669. doi:10.1007/s00381-005-0020-4 [80]	Childs Nervous System	2006
71	121	Egnor M, Zheng LL, Rosiello A, Gutman F, Davis R. A model of pulsations in communicating hydrocephalus. Article. Pediatric Neurosurgery. Jun 2002;36(6):281-303. doi:10.1159/000063533 [81]	Pediatric Neurosurgery	2002
72	120	Alvarez LA, Maytal J, Shinnar S. Idiopathic external hydrocephalus – natural-history and relationship to benign familial macrocephaly. Article. Pediatrics. Jun 1986;77(6):901-907 [82]	Pediatrics	1986
73	119	Oi S, Shimoda M, Shibata M, et al. Pathophysiology of long-standing overt ventriculomegaly in adults. Article. Journal of Neurosurgery. Jun 2000;92(6):933-940. doi:10.3171/jns.2000.92.6.0933 [83]	Journal of Neurosurgery	2000
74	119	Baskin JJ, Manwaring KH, Rekate HL. Ventricular shunt removal: the ultimate treatment of the slit ventricle syndrome. Article. Journal of Neurosurgery. Mar 1998;88(3):478-484. doi:10.3171/jns.1998.88.3.0478 [84]	Journal of Neurosurgery	1998
75	119	Raftopoulos C, Deleval J, Chaskis C, et al. Cognitive recovery in idiopathic normal-pressure hydrocephalus – a prospective study. Article. Neurosurgery. Sep 1994;35(3):397-404. doi:10.1227/00006123-199409000-00006 [85]	Neurosurgery	1994
76	118	Tulipan N, Wellons JC, Thom EA, et al. Prenatal surgery for myelomeningocele and the need for cerebrospinal fluid shunt placement. Article. Journal of Neurosurgery-Pediatrics. Dec 2015;16(6):613-620. doi:10.3171/2015.7.Peds15336 [86]	Journal of Neurosurgery-Pediatrics	2015
77	117	Vinchon M, Dhellemmes P. Cerebrospinal fluid shunt infection: risk factors and long-term follow-up. Article. Childs Nervous System. Jul 2006;22(7):692-697. doi:10.1007/s00381-005-0037-8 [87]	Childs Nervous System	2006
78	117	Boon AJW, Tans JTJ, Delwel EJ, et al. Dutch normal-pressure hydrocephalus study: the role of cerebrovascular disease. Article. Journal of Neurosurgery. Feb 1999;90(2):221-226. doi:10.3171/jns.1999.90.2.0221 [88]	Journal of Neurosurgery	1999
79	115	Sheehan JP, Polin RS, Sheehan JM, Baskaya MK, Kassell NF. Factors associated with hydrocephalus after aneurysmal subarachnoid hemorrhage. Article. Neurosurgery. Nov 1999;45(5):1120-1127. doi:10.1097/00006123-199911000-00021 [89]	Neurosurgery	1999
80	114	Warf BC, Campbell JW. Combined endoscopic third ventriculostomy and choroid plexus cauterization as primary treatment of hydrocephalus for infants with myelomeningocele: long-term results of a prospective intent-to-treat study in 115 East African infants. Article. Journal of Neurosurgery-Pediatrics. Nov 2008;2(5):310-316. doi:10.3171/ped.2008.2.11.310 [90]	Journal of Neurosurgery-Pediatrics	2008
81	114	Casey ATH, Kimmings EJ, Kleinlugtebeld AD, Taylor WAS, Harkness WF, Hayward RD. The long-term outlook for hydrocephalus in childhood - a ten-year cohort study of 155 patients. Article. Pediatric Neurosurgery. Aug 1997;27(2):63-70. doi:10.1159/000121229 [91]	Pediatric Neurosurgery	1997
82	113	Drake JM, Canadian Pediat Neurosurg Study G. Endoscopic third ventriculostomy in pediatric patients: the Canadian experience. Article. Neurosurgery. May 2007;60(5):881-885. doi:10.1227/01.Neu.0000255420.78431.E7 [92]	Neurosurgery	2007
83	113	Marmarou A, Foda MAA, Bandoh K, et al. Posttraumatic ventriculomegaly: hydrocephalus or atrophy? A new approach for diagnosis using CSF dynamics. Article. Journal of Neurosurgery. Dec 1996;85(6):1026-1035. doi:10.3171/jns.1996.85.6.1026 [93]	Journal of Neurosurgery	1996
84	113	Nagashima T, Tamaki N, Matsumoto S, Horwitz B, Seguchi Y. Biomechanics of hydrocephalus – a new theoretical model. Article. Neurosurgery. Dec 1987;21(6):898-904. doi:10.1227/00006123-198712000-00019 [94]	Neurosurgery	1987
85	113	Milhorat TH. Acute hydrocephalus after aneurysmal subarachnoid hemorrhage. Article. Neurosurgery. Jan 1987;20(1):15-20. doi:10.1227/00006123-198701000-00004 [95]	Neurosurgery	1987
86	110	Tulipan N, Sutton LN, Bruner JP, Cohen BM, Johnson M, Adzick NS. The effect of intrauterine myelomeningocele repair on the incidence of shunt-dependent hydrocephalus. Article. Pediatric Neurosurgery. Jan 2003;38(1):27-33. doi:10.1159/000067560 [96]	Pediatric Neurosurgery	2003
87	110	Black PM. Hydrocephalus and vasospasm after subarachnoic hemorrhage from ruptured intracranial aneurysms. Article. Neurosurgery. Jan 1986;18(1):12-15. doi:10.1227/00006123-198601000-00003 [97]	Neurosurgery	1986
88	109	O'Brien DF, Javadpour M, Collins DR, Spennato P, Mallucci CL. Endoscopic third ventriculostomy: an outcome analysis of primary cases and procedures performed after ventriculoperitoneal shunt malfunction. Article. Journal of Neurosurgery. Nov 2005;103(5):393-400. doi:10.3171/ped.2005.103.5.0393 [98]	Journal of Neurosurgery	2005
89	108	Klinge P, Marmarou A, Bergsneider M, Relkin N, Black PM. Outcome of shunting in idiopathic normal-pressure hydrocephalus and the value of outcome assessment in shunted patients. Article. Neurosurgery. Sep 2005;57(3):40-52. doi:10.1227/01.Neu.0000168187.01077.2f [99]	Neurosurgery	2005
90	108	Koch D, Wagner W. Endoscopic third ventriculostomy in infants of less than 1 year of age: which factors influence the outcome? Article. Childs Nervous System. Jun 2004;20(6):405-411. doi:10.1007/s00381-004-0958-7 [100]	Childs Nervous System	2004
91	107	Robinson S. Neonatal posthemorrhagic hydrocephalus from prematurity: pathophysiology and current treatment concepts. Review. Journal of Neurosurgery-Pediatrics. Mar 2012;9(3):242-258. doi:10.3171/2011.12.Peds11136 [101]	Journal of Neurosurgery-Pediatrics	2012
92	107	Kulkarni AV, Drake JM, Armstrong DC, Dirks PB. Imaging correlates of successful endoscopic third ventriculostomy. Article. Journal of Neurosurgery. Jun 2000;92(6):915-919. doi:10.3171/jns.2000.92.6.0915 [102]	Journal of Neurosurgery	2000
93	107	Sainte-Rose C, Hooven MD, Hirsch JF. A new approach in the treatment of hydrocephalus. Article. Journal of Neurosurgery. Feb 1987;66(2):213-226. doi:10.3171/jns.1987.66.2.0213 [103]	Journal of Neurosurgery	1987
94	107	Engel M, Carmel PW, Chutorian AM. Increased intra-ventricular pressure without ventriculomegaly in children with shunts – normal volume hydrocephalus. Article. Neurosurgery. 1979;5(5):549-552. doi:10.1227/00006123-197911000-00001 [104]	Neurosurgery	1979
95	106	Di Rocco C, Massimi L, Tamburrini G. Shunts vs endoscopic third ventriculostomy in infants: are there different types and/or rates of complications? A review. Review. Childs Nervous System. Dec 2006;22(12):1573-1589. doi:10.1007/s00381-006-0194-4 [105]	Childs Nervous System	2006
96	106	Beems T, Grotenhuis JA. Is the success rate of endoscopic third ventriculostomy age-dependent? An analysis of the results of endoscopic third ventriculostomy in young children. Article. Childs Nervous System. Nov 2002;18(11):605-608. doi:10.1007/s00381-002-0652-6 [106]	Childs Nervous System	2002
97	106	Bogdahn U, Lau W, Hassel W, et al. Continuous-pressure controlled, external ventricular drainage for treatment of acute hydrocephalus – evaluation of risk-factors. Article. Neurosurgery. Nov 1992;31(5):898-904. doi:10.1227/00006123-199211000-00011 [107]	Neurosurgery	1992
98	105	Papile LA, Burstein J, Burstein R, Koffler H, Koops BL, Johnson JD. Post-hemorrhagic hydrocephalus in low-birth-weight infants – treatment by serial lumbar punctures. Article. Journal of Pediatrics. 1980;97(2):273-277. doi:10.1016/s0022-3476(80)80494-x [108]	Journal of Pediatrics	1980
99	104	Stein SC, Guo WS. Have we made progress in preventing shunt failure? A critical analysis. Review. Journal of Neurosurgery-Pediatrics. Jan 2008;1(1):40-47. doi:10.3171/ped-08/01/040 [109]	Journal of Neurosurgery-Pediatrics	2008
100	104	Albright AL, Haines SJ, Taylor FH. Function of parietal and frontal shunts in childhood hydrocephalus. Article. Journal of Neurosurgery. Dec 1988;69(6):883-886. doi:10.3171/jns.1988.69.6.0883 [110]	Journal of Neurosurgery	1988

Type and Field of Study

Articles were also distinguished by study type, as indicated by PubMed or evidenced in the article, and further stratified by etiology. If the etiology was not specified, studies were instead subcategorized by treatment type (Table 3). Pediatric studies (n=14) included those specific to the pediatric population and were separated from articles concerning infants (n=9) and pediatric studies not further classified by etiology. Etiologies such as aqueductal stenosis, tumors, and other obstructive causes of hydrocephalus were classed as obstructive (n=6). Communicating (n=15) included idiopathic, normal pressure hydrocephalus, and other non-obstructive etiologies. The category “other” (n=3) was assigned to studies that included etiologies, populations, and/or treatments that did not fit into the classifications previously outlined. Of the 100 articles referenced, 38 were review articles, 24 were original articles, 15 were comparative studies, 11 were clinical trials, six were multi-center studies, three were cross-sectional, and three were case reports with reviews. Original prospective non-review articles and retrospective reviews accounted for more than half of the selected articles. Posthemorrhagic and communicating hydrocephalus were the most commonly cited by etiology (n=15). Concerning treatment, the most frequently cited subcategory was shunt-related articles (n=19). Comparative studies and clinical trials were most frequent in articles relating to shunts (n=4 and n=5, respectively). Cross-sectional studies were most common in articles specific to pediatrics (n=2) and multicenter studies (n=4). Case reports appeared most often in relation to posthemorrhagic studies (n=2).

**Table 3:** Type and field of study

Type and Field of Study											
Study Type	Infantile	Pediatric	Post-hemorrhagic	Communicating	Obstructive	Neural tube defect-related	Post-infectious	Other	Shunt	Ventriculostomy	Total
Case report	0	0	2	0	1	0	0	0	0	0	3
Comparative study	2	2	2	2	1	1	0	0	4	1	15
Clinical trial	1	2	0	1	0	1	0	1	5	0	11
Cross-sectional	0	2	0	0	0	0	0	0	0	1	3
Multi-center study	0	4	0	0	0	0	0	0	1	1	6
Original	3	1	5	6	1	2	2	1	3	1	24
Review	3	3	5	6	3	1	0	1	10	4	38
Total	9	14	15	15	6	5	2	3	19	8	100

Discussion

Hydrocephalus represents a significant burden of disease, as substantiated by the estimated regional incidence in 2018 of 68 per 100,000 births in the US/Canada and the more than 383,000 new cases annually worldwide [111]. Its epidemiological impact translates to the clinical realm, heightening the urgency for understanding and managing hydrocephalus. From a neurosurgical standpoint, hydrocephalus is one of the most frequently treated conditions in the field, and its impact is great [30]. Given this, a centralized archive of pertinent literature is of great clinical value. Consistent with several new publications focusing on highly-cited literature within neurosurgery, we aimed to generate a list of the most relevant works on hydrocephalus published during the last four and a half decades [6,7]. Using bibliometric analysis, we created a catalog of the 100 most cited publications specific to hydrocephalus literature. We then further subcategorized our results by journal, article type, publication year, and number of citations. By analyzing highly cited journal articles, we have outlined critical reference sources and highlighted important trends throughout the literature. The exclusion of articles unrelated to hydrocephalus, those based on basic science research, and those including animal studies maintained our intention to consider the scope of hydrocephalus in the context of clinical neurosurgery. With this aim, we focused on different etiologies of this pathology alongside the surgical or medical management of hydrocephalus.

Ponce and Lozano demonstrated the importance of recognizing highly-cited literature in neurosurgery overall [6]. Wilcox et al. and, more recently, Grayson et al. extended their work and continued to focus on broader areas of study such as neurosurgery and, alternatively, pediatric neurosurgery [7,112,113]. The feasibility of this form of analysis can also be demonstrated with a more targeted focus on conditions and treatments frequently encountered in neurosurgery and neurosurgical research. The field of citation analysis has grown over the past decade since Ponce and Lozano originally published their article and has grown to include many topics within the field of neurosurgery, such as trigeminal neuralgia, stereotactic radiosurgery for meningioma, and spinal deformity [114-116]. There are multiple ways of interpreting the data, including by country, institution, and authorship. The impact factor of selected journals and the number of highly cited works in each journal were also analyzed, with 88% of highly cited papers being found in journals with an impact factor greater than 1. Multiple authors in the literature have debated the importance of impact factors, with journals assigned higher impact factors believed to attract papers of higher quality. This may not reflect the quality of the peer review process or the content of the journal [117,118]. It has also been proposed that journals publishing more review articles achieve higher impact factors due to the nature of review papers being cited more frequently than other types of research articles [119]. This was also seen in our literature review, with 33% of highly cited papers being reviews compared to 24% of original articles and 15% of comparative studies being the other highly cited journal article categories.

The list of most cited works in descending order of citation number is led by a randomized trial published in 1998 with 469 citations to assess the design of CSF shunt valves in pediatric hydrocephalus. Published in Neurosurgery, this article holds relevance for neurosurgeons, neurosurgical trainees, neuroradiologists, pediatricians, and any student of the neuroanatomical relationship that gives rise to the pathophysiology of hydrocephalus. When viewing the works listed in this manner, it may be beneficial to future surveyors of neurosurgical publications to analyze trends between top-cited lists. Such reviews may compare top-cited works on hydrocephalus, pediatric neurosurgery, and/or other pertinent pediatric neurosurgical pathology to understand how the most relevant works have molded current practices and guided the knowledgebase of today’s neurosurgical trainees. With our top 10 most cited papers being published between 1985 and 2005, nearly two decades ago, this type of comparative review of relevant works over time would provide an interesting and informative direction for future similar pieces in the field. As the complexity of this pathology often requires a multidisciplinary care approach to provide the best long-term outcomes for these patients, this method of analysis will better serve the neurosurgical teams and other clinical subspecialties in the care of these patients. By establishing the 100 most cited hydrocephalus articles, we contribute one source, stratified for efficient referencing, to facilitate the exchange of knowledge, clinical care, and future research on hydrocephalus.

Limitations

Citation analysis is not without limitations; the correlation between the number of citations and the impact of a journal article has been debated [5]. The journals selected for analysis do not cover all journals in which highly cited articles can be published, and high-impact articles could have been missed. Owing to the time bias of citations, more recent papers have had less time to accumulate citations than older articles [120]. This sum of works is thus not a true rank list but rather a collection of the greatest hits. There are also limitations within the database used to research citations; the Web of Science Core Collection only includes articles from 1976 to 2021, so earlier influential journal articles are unlikely to be well represented within the search. Other databases, such as Microsoft Academic and Google Scholar, contain more citations than the Web of Science Core collection, so they could be alternative sources for citation data [4].

## Conclusions

This study identified the 100 most cited journal articles on hydrocephalus from journals selected using the Journal Citation Reports database. Bibliometric analysis using the Web of Science Core Collection was used to identify the highest-impact articles based on the number of reported citations. While this analysis does not guarantee flawless determination of the impact and quality of publications, it allows published data to be easily referenced so that clinicians and researchers can identify meaningful research and cases in the study and treatment of hydrocephalus.
